# Xenon lamps used for fruit surface sterilization can increase the content of total flavonols in leaves of *Lactuca sativa* L. without any negative effect on net photosynthesis

**DOI:** 10.1371/journal.pone.0223787

**Published:** 2019-10-21

**Authors:** Salah Fgaier, Mônica Maria de Almeida Lopes, Ebenézer de Oliveira Silva, Jawad Aarrouf, Laurent Urban

**Affiliations:** 1 UMR 95 Qualisud/Laboratoire de Physiologie des Fruits et Légumes, Avignon Université, Avignon, France; 2 NOVAGENETIC, Anjou Actiparc, Longué Jumelle, France; 3 Embrapa Agroindústria Tropical, Rua Dra, Sara Mesquita, CEP, Fortaleza, CE, Brazil; National Research Council of Italy, ITALY

## Abstract

One (1P), two (2P), three (3P) or four (4P) pulses of light supplied by a xenon lamp, were applied to young lettuce plants grown in pots. The lamp used in the trial was similar to those used for fruit surface sterilization. Total flavonols were measured in leaves using the Dualex method. In a first trial conducted in greenhouse conditions, 6 days after the pulsed light (PL) treatment, flavonols were increased by 312% and 525% in the 3P and 4P treatments, respectively, in comparison to the those in the untreated control. Changes in the chlorophyll fluorescence parameters suggest that the PL treatment may induce limited and transient damage to the photosynthetic machinery and that the damage increases with the increasing number of pulses. The performance parameters were not significantly affected by PL and recovered fully by 6 days after the treatments. The 1P and the 2P treatments 6 days after the treatment showed a 28.6% and a 32.5% increase, respectively, in net photosynthetic assimilation, when compared to that of the control. However, 8 days after the treatment, there was no longer a difference between the treatments and the control in net photosynthetic assimilation. Eight days after the light treatment, the 3P treatment showed a 38.4% increase in maximal net photosynthetic assimilation over that of the control, which is an indication of positive long-term adaptation of photosynthetic capacity. As a whole, our observations suggest that PL could be used on field or greenhouse crops to increase their phytochemical content. No long-lasting or strong negative effects on photosynthesis were associated with PL within the range of doses we tested; some observations even suggest that certain treatments could result in an additional positive effect. This conclusion is supported by a second trial conducted in phytotrons. More studies are required to better understand the roles of the different wavelengths supplied by PL and their interactions.

## Introduction

Pulsed light (PL) is provided by xenon or xenon-mercury lamps and supplies high-intensity light, in the 185 to 2000 nm range, which encompasses UV radiation, notably UV-C radiation (200–280 nm), radiation in the visible domain and near infrared radiation. Based on the decontaminating properties of UV radiation, PL was developed for surface sterilization purposes and is currently used in the medical field [1] and in the food industry [2]. The disinfectant properties of PL can be beneficially used not only on inert surfaces, but also on fruits and vegetables after harvest, for instance, to potentially extend their shelf- life [3,4]. The content of secondary metabolites, so-called phytochemicals, was analyzed in treated fruits after several days of storage with the objective of verifying that PL, at doses that are effective for disinfection, does not cause any negative effects to the contents of health-promoting compounds such as vitamin C, phenolic compounds, or carotenoids. It was found that it is possible to define hormetic doses of PL for stored fruits, i.e., doses that have the desired effect (disinfection), but that are at the same time harmless as far as phytochemical contents are concerned [5–9]. Moreover, it was found that it is possible to define doses that are capable of literally increasing over several days the phytochemical contents of fruits subjected to PL at the beginning of their storage period.

Now that postharvest studies have revealed the potential of PL to increase the production of phytochemicals in harvested organs, it seems tempting to test the potential of PL before harvest to stimulate the production of phytochemicals and plant natural defenses [10–12].

While photosynthesis is not believed to play a significant role in fruit and vegetable preservation after harvest, photosynthesis is pivotal for crop performance in the field. The potential impact of high levels of UV-B light has been extensively studied as a part of global change studies. See, for instance, [13]. Later, [14], refuting numerous observations about the negative effects of UV-B light, concluded that realistic doses of UV-B light do not represent a real threat to photosynthesis, plant growth or crop productivity. However, PL supplies UV light in the form of high-intensity flashes, and it also supplies UV-C radiation, which can have strong inhibiting and even damaging effects on the photosynthetic machinery [15–18]. Even flashes of light in the visible range are able to induce D1 protein degradation [19] or to damage the oxygen-evolving complex [20]. It is therefore essential to determine whether the doses of PL that are effective for stimulating secondary metabolism, negatively impact photosynthesis.

The objective of this study was to test the effects of different doses of PL on the production of total flavonols and hydroxycinnamic acids in the leaves of lettuce plants grown in greenhouse conditions, and to check whether the doses that are effective for stimulating the accumulation of flavonols or hydroxycinnamic acids, negatively impact photosynthesis. To the best of our knowledge, this is the first trial of the effect of PL at the whole-plant level, not just on a harvested organ, and most importantly focused on photosynthetic machinery. PL in our trial was supplied by a system that can be operated at 220 V instead of 380 V, and could be adapted to field conditions. Otherwise this system is similar to the ones used for fruit surface sterilization. In addition to net photosynthesis, we measured maximal photosynthesis, which is related to photosynthetic capacity, and different parameters derived from chlorophyll fluorescence (ChlF) measurements that are indicators of potential damage to the photosynthetic machinery or to major adaptative processes of rerouting of electron and energy fluxes [21]. Flavonols and hydroxycinnamic acids were chosen as examples of secondary metabolites. Flavonols have health-promoting properties and are involved in plant responses against biotic and abiotic stresses; they are easy to measure in leaves using nondestructive chlorophyll fluorescence-based methods [22]. A second independent trial was conducted in growth chambers. In this second trial, we focused on flavonols and the major ChlF parameters only.

## Materials and methods

### Plant material and experimental design

**Fig 1:**
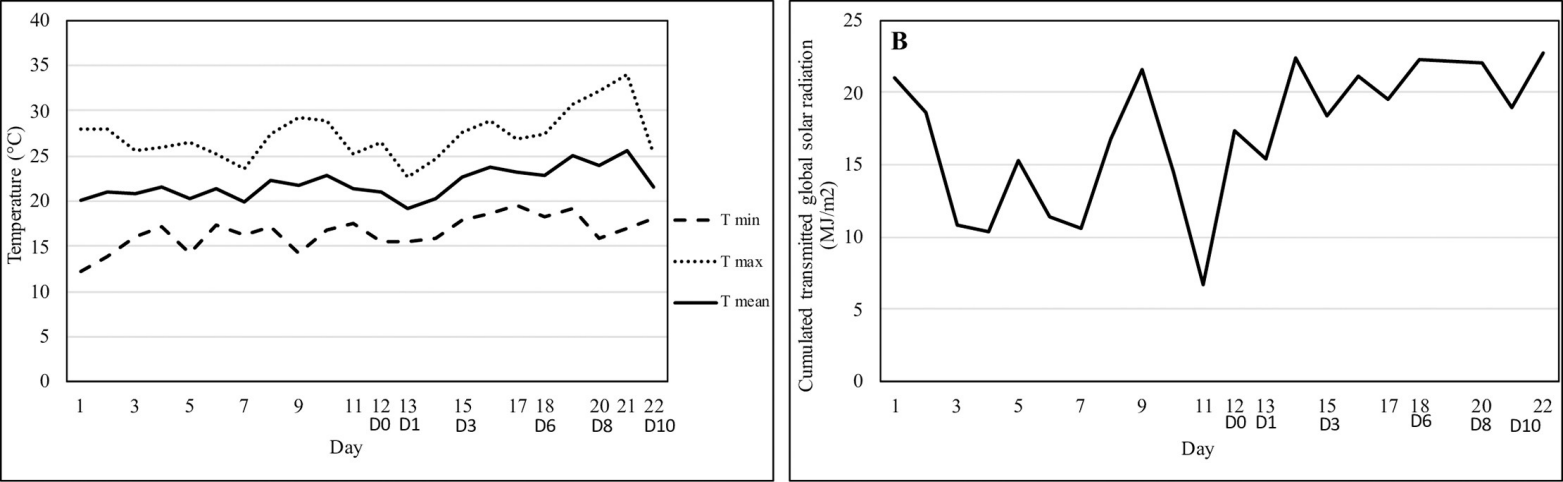
Daily mean maximum and minimum temperatures (A), and daily cumulated transmitted global solar radiation (B) at trial one. D0 corresponds of PL treatments.

Trial one (2018):

The first trial was conducted in the greenhouse facilities of Avignon University (France). The daily mean, maximum and minimum temperatures and daily cumulated transmitted global radiation are given in Fig 1A and 1B. Lettuce seeds (*Lactuca sativa* L. cv Joviale) were sown in seedling plates for one week at 25°C ±2°C. Then, seedlings were transplanted into pots (9 cm in diameter) and raised for 15 days in greenhouse conditions. At the time of measurement, the temperature was above the growth temperature because of exceptionally high temperatures at that time of the year (Fig 1A). The substrate was a typical horticulture mixture (Klasmann Deilmann Gmbh, Bremen, Germany) containing 80% organic matter, at pH 6. A regular water regime was applied for all of the plants every two days. We used a fertilizer with the following composition: 5% N, 5% P_2_O_5_, 7% K_2_O, 2.5% MgO, 12% SO_3_ and 13% CaO. Ten control plants and 10 plants for each of the four PL treatments were randomly distributed in the greenhouse.

Trial two (2019):

A second trial was conducted in growth chambers (Bionef, France) located at Avignon University (France). Lettuce seeds (*Lactuca sativa* L. cv. Joviale) were sown in plates at 25°C ±2°C. After one week, seedlings were transplanted into pots (9 cm in diameter) and raised for 15 days in controlled conditions (25°C day/22°C night; 16 h day/8 h night). The light intensity was set at 300 μmol photons.m^-2^.s^-1^. Substrate, fertilization and irrigation were similar to those in trial one. Ten control plants and 10 plants for each of the four PL treatments were randomly distributed in the growth chamber.

### The pulsed light system

**Fig 2:**
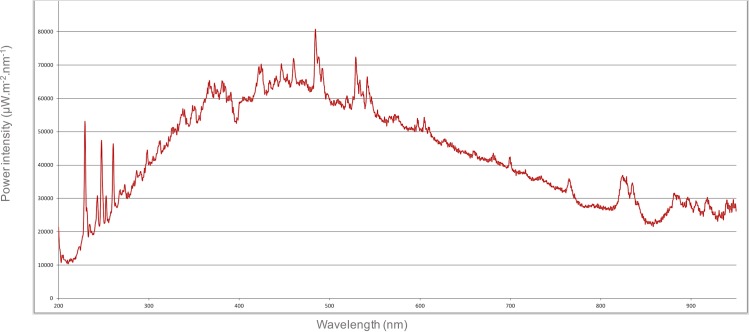
Typical spectrum of pulsed light (PL) provided by Phoxène-Lumix, Dardilly, France.

The PL system consisted of a FX-DB xenon lamp (Phoxène-Lumix S.R.L., Dardilly, France), capable of supplying 0.8 J cm^-2^ in 500 μs on a 50 cm^2^ surface at a distance of 5 cm. The energy dose was measured using a Joulemeter Integra detector (Gentec Electro-optics Inc., Québec city, Canada). The PL system of Phoxène-Lumix is different from other existing systems because it can be operated using 220 instead of 380 V. Fig 2 shows a typical PL spectrum provided by Phoxène-Lumix. Plants were subjected to PL in a box specifically designed to accommodate plants for treatment while ensuring that users could not be exposed to unwanted radiation. Treatments were performed on the top of the rosettes. PL treatments were performed on June 12^th^, 2018 (D0). Treatments were derived from the standard procedures for fruit surface sterilization; they consisted of either one (1P), two (2P), three (3P) or four (4P) successive pulses, each of 500 μs, separated by periods of 15 s.

### Measurements of leaf gas exchange

The net CO_2_ assimilation rate (A_net_) and leaf stomatal conductance of water vapor (g_s_) were measured every two days, between 10 am and noon, using an infrared CO_2_/H_2_O gas analyzer and leaf chamber system with an external light source in the 400–700 nm range (LI 6800, Li-Cor, Lincoln, NE). Photosynthetic photon flux density (PPFD) was set at 500 μmol photons m^– 2^ s^–1^ and partial pressure of ambient CO_2_ (C_a_) at 40 Pa. Leaf temperature was not controlled for the sake of measurement speed, and ranged from 30.5 to 33.8°C. In addition to A_net_, we calculated g_s_ and the internal partial pressure of CO_2_ (C_i_).

The maximal rate of net photosynthesis in conditions of nonlimiting photon flux density and CO_2_ (A_max_) was measured at the end of the trial, as an indicator of photosynthetic capacity [23]. For A_max_ measurements, PPFD was set at 1500 μmol photons m^– 2^ s^–1^ and C_a_ at 200 Pa. Single leaf gas exchange measurements were generally less than 2 mn.

Leaf gas exchange measurements were made in trial one only. For all gas exchange measurements, n = 10.

### Measurements of ChlF and calculation of the parameters derived from ChlF induction curves

Chlorophyll *a* fluorescence transients were measured on leaves different from the leaves used for gas exchange measurements, before 10 am, with a Pocket PEA chlorophyll fluorimeter (Hansatech Instruments, King’s Lynn, Norfolk, United Kingdom). Leaves were dark-adapted for 20 minutes with a lightweight plastic leaf clip prior to measuring. The transients were induced by 1 s illumination with a single light-emitting diode providing a fully saturating photon flux density of 3500 μmol photons m^– 2^ s^–1^ with a peak wavelength of 627 nm at the sample surface, and homogeneous irradiation. The ChlF intensity at 50 μs was considered as F_0_ [24]. The fast ChlF kinetics (from F_0_ to F_m_, where F_0_ and F_m_ were, respectively, the minimum and maximum measured chlorophyll fluorescence of PSII in the dark-adapted state) were recorded from 10 μs to 1 s. As described in [25], the maximum quantum yield of photosystem II (PSII), the ratio of variable ChlF (F_v_) to maximum ChlF (F_m_), (F_v_/F_m_), the performance index (PI), a plant vitality indicator [26] and their components (F_v_/F_0_, RC/ABS which represents the ratio of reaction centers to the absorbance, (1–V_j_)/V_j_) where V_j_ is the relative variable ChlF at time J = 2 ms) were calculated automatically. We also calculated the dissipated energy flux per PSII reaction centers (DI_0_/RC), an indicator of the importance of processes other than trapping, and the electron transport flux from Q_B_ to PSI acceptors, RE_0_, expressed as quantum yield (/ABS) which is arguably related to cyclic electron transport (CET) activity [21]. Changes in CET activity play a major role in plant adaptation to stress.

We calculated the following parameters which are indicators of potential damage: F_0_, F_v_/F_m_, V_k_/V_j_ and S_m_ [21]. V_k_/V_j_ represents the ratio of variable ChlF at 300 μs (K-step) to variable ChlF at 2 ms (J-step), and S_m_ is the normalized area above the ChlF induction curve.

ChlF was measured in trials one and two. For all measurements of ChlF using the Pocket PEA, n = 20 (two leaves per plant).

### Measurements of total flavonols and hydroxycinnamic acids in the epidermis of lettuce leaves

To evaluate the contents of total hydroxycinnamic acids (trial one) and total epidermal flavonols (trials one and two), we used nondestructive techniques based on the ChlF excitation ratio method [27–30]. We used the Dualex HCA for hydroxycinnamic acid contents and the Dualex Flav Force-A (Orsay, France) for flavonol contents. The latter also takes measurements of chlorophyll by transmittance and provides an index for anthocyanins.

Following [28,30], the flavonoid index serving as an estimate of UV-absorbing compounds (at 375 nm), mostly flavonols, was calculated as the logarithm of the ratio of red-light induced far-red ChlF to UV-induced far-red ChlF. We did not use the modified flavonoid index proposed by [22] for lettuce since anthocyanins were nearly absent in the leaves and did not contribute to any screening effect at 375 nm.

For all measurements of ChlF using the Dualex systems, n = 20 (two leaves per plant). Each measurement per leaf was the mean of three measurements taken on the upper surface, avoiding major veins.

### Statistics

For each measurement date of the different measured parameters, the Kruskal-Wallis nonparametric statistical test was applied. When the difference was significant between the treatments at the same measurement date, a multiple comparison with the Dunnett test was performed. All statistical analyses were performed using R software.

## Results

### Effect of PL on the contents of chlorophyll, total flavonols and total hydroxycinnamic acids

**Fig 3:**
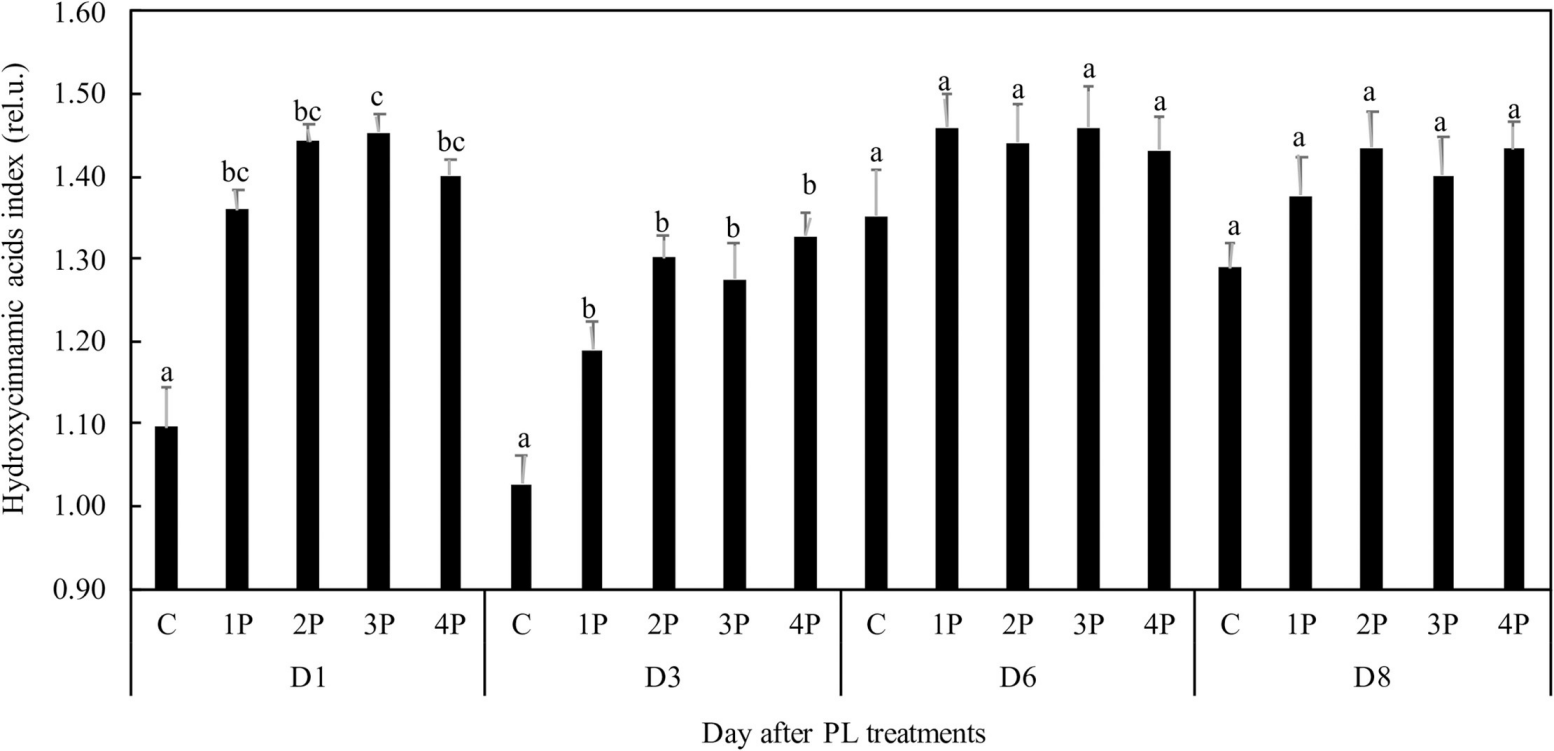
Effect of PL treatments (1P, 2P, 3P, 4P) on the hydroxycinnamic acids index.

**Fig 4:**
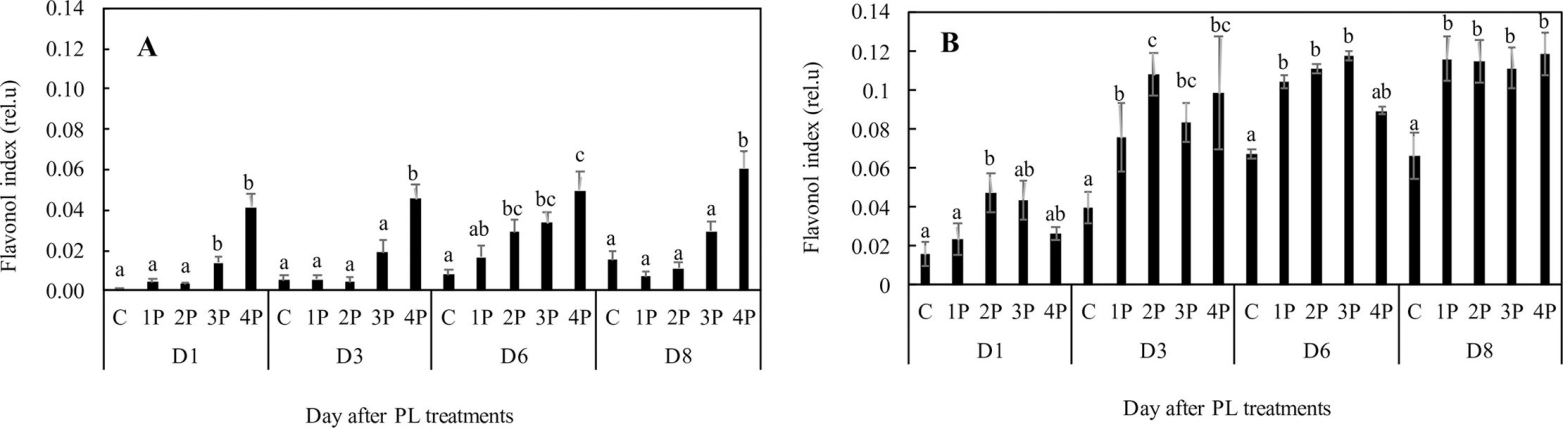
Effects of PL treatments (1P, 2P, 3P, 4P) on the flavonol index measured during trial one (A) and trial two (B).

Chlorophyll was found to be slightly increased in trial one at D1 (1 day after PL treatments), by 1.4% and by 2.9% in the 3P and 4P treatments, respectively, compared to that of the control. There were no differences after D1, with the exception of 2P at D8 (8 days after PL treatments). On that date, the increase was 9.6% compared to that of the control (data not shown). On the first day after the treatment, the hydroxycinnamic acid content was higher in all treatments in trial one, and the increase was up to 39.6% (3P) in comparison with that of the control. This difference was still significant at D3. After D3, the difference from the control was no longer significant (Fig 3). Total flavonols in the epidermis were found to be higher in trial one 6 days after the treatment (D6), by 312% and 525% in the 3P and 4P treatments, respectively, compared to that of the control. The increase became apparent as soon as D3 in the 3P and 4P treatments (Fig 4A). The stimulating effect of PL on total flavonols in the epidermis was confirmed in trial two. The difference from that in the control was significant only in the 2P treatment at D1 (+ 198%), but there was an average 74% increase in all PL treatments compared to that of the control at D8 (Fig 4B).

### Effect of PL on damage parameters derived from induction curves of chlorophyll fluorescence

**Fig 5:**
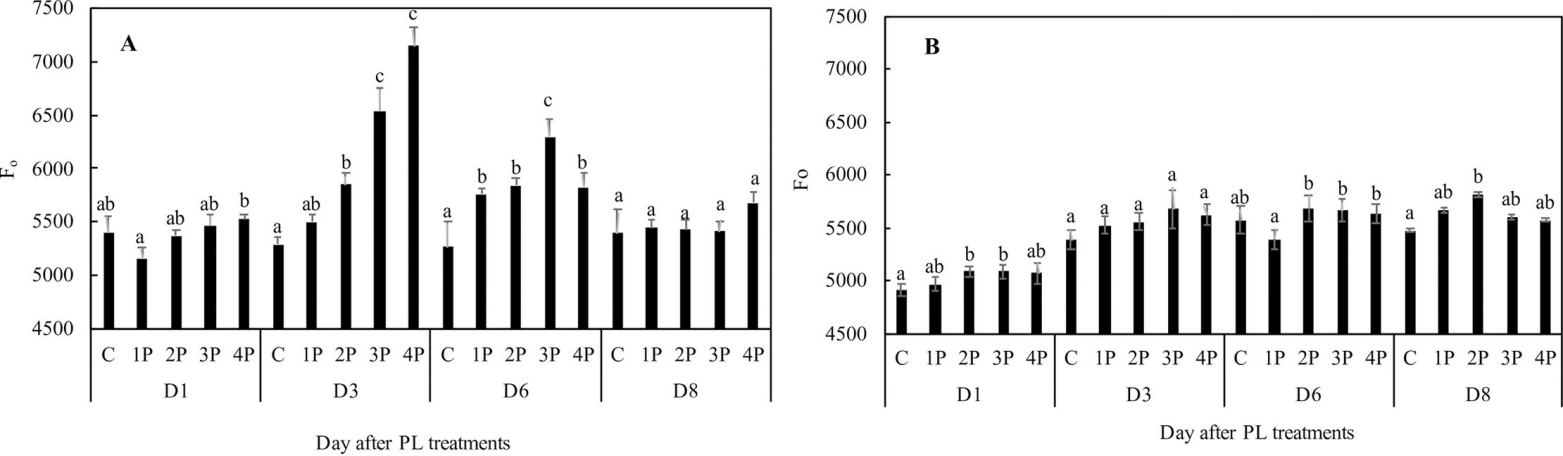
Effects of PL treatments (1P, 2P, 3P, 4P) on minimum of fluorescence measured during trial one (A) and trial two (B).

Increases in F_0_ were observed in trial one as a consequence of the PL treatments on 12 June as soon as the day after for the 4P treatment, but the most marked effects when compared to the control were observed at D3 for the 4P treatment (35.1%), and they were also observable for the 3P (23.6%) and the 2P treatments (10.6%) on that date. A modest increase in F_0_ over that of the control for the 1P treatment was observed only at D6 (9.3%). At D8, there were no more significant observable differences from the control (Fig 5A).

**Fig 6:**
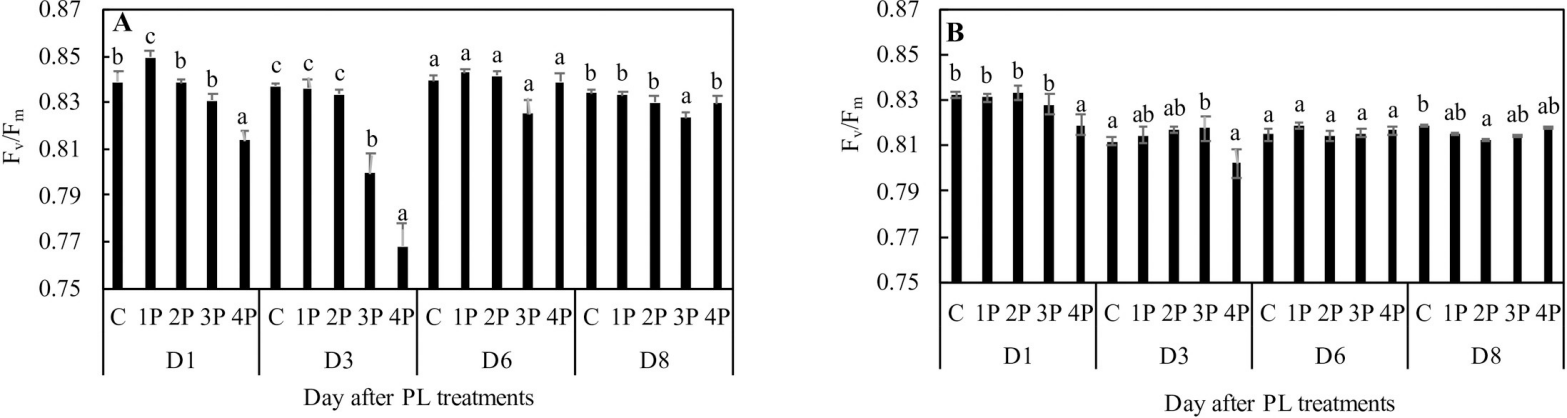
Effects of PL treatments (1P, 2P, 3P, 4P) on the maximum quantum yield of primary PSII chemistry (F_v_/F_m_) measured during trial one **(A)** and trial two **(B)**.

Not all increases in F_0_ translated into decreases in F_v_/F_m_ which is to be expected since the latter may also be due to decreases in F_m_. An increase in F_m_ can counteract the negative effect of an increase in F_0_ on F_v_/F_m_. However, consistent with the F_0_ data, the most marked decreases in F_v_/F_m_ were observed for the 3P and 4P treatments at D3 in trial one: 4.5% and 8.2%, respectively, compared to that of the control. There were no differences at D8 with the exception of the 3P treatment, but that F_v_/F_m_ decrease from that of the control was very small (1.3%) (Fig 6A). The two fluorescence parameters, F_0_ and F_v_/F_m_, showed slightly different behavior in trial two (Figs 5B and 6B). F_v_/F_m_ was slightly lower (1.6%) in the 4P treatment than that of the control at D1 and in the 2P treatment at D8 (0.8%), whereas F_0_ was 3.7% higher than that of the control in the 2P and 3P treatments at D1, and 6% higher than that of the control in the 2P treatment at D8 (Figs 5B and 6B).

**Fig 7:**
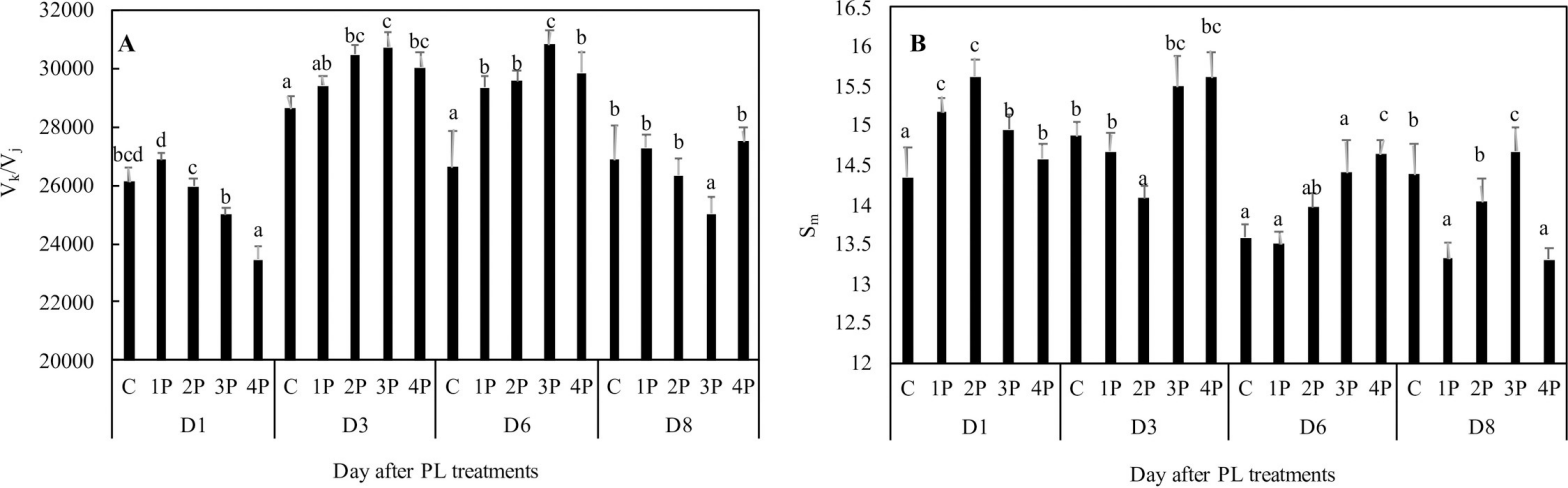
Effects of PL treatments (1P, 2P, 3P, 4P) on damage indicators. The measurements were made 1, 3, 6 or 8 days after PL treatments (Trial one). V_k_/V_j_ is an indicator of inactivation of the oxygen evolving complex **(A)** and S_m_ the normalized area above the OJIP curve **(B)**.

In trial one, the increase in V_k_/V_j_ compared to that of the control ranged from 10.3% (1P) to 15.8% (3P) at D6. This increase was already observable in the 2P, 3P and 4P treatments (not significantly for the latter) at D3 (Fig 7A). At D8, all treatments had recovered with the exception of the 3P treatment. In trial two, full recovery was observed for all treatments at D8 without exception (data not shown).

The pattern for S_m_ was more complex. In trial one, small decreases in S_m_, of 7.4% and 7.5%, were observed at D8 for the 1P and 4P treatments, respectively, compared to that of the control. A decrease of 5.3% was observed for the 2P treatment as early as D3, but the difference from the control was no longer significant at D8. S_m_ was never found to be lower in the 3P treatment than in the control on any of the measurement dates (Fig 7B). In trial two, PL treatments similarly never resulted in decreases in S_m_. In contrast, S_m_ was higher in the 3P treatment than in the control at D8 (data not shown).

**Fig 8:**
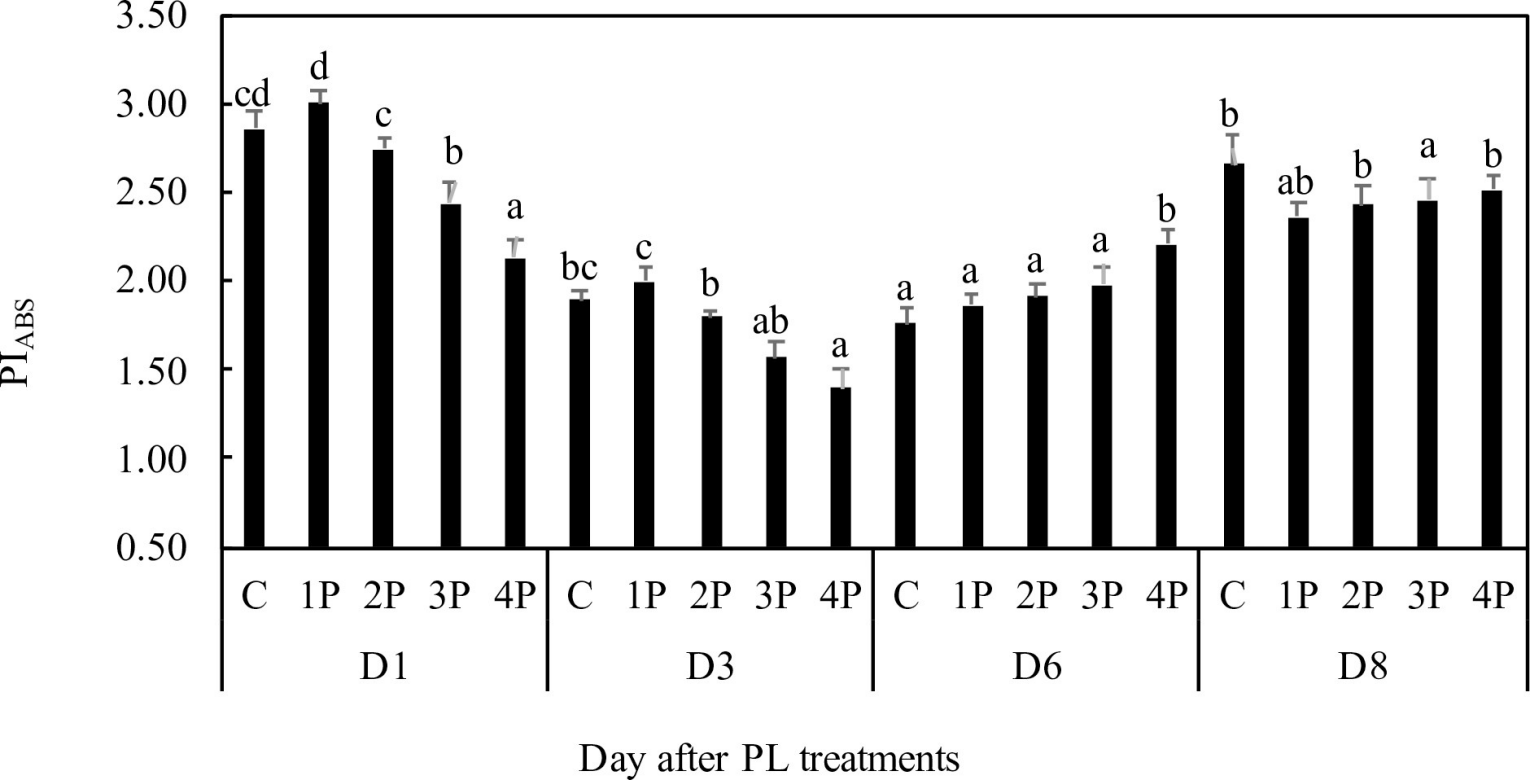
Effects of PL treatments (1P, 2P, 3P, 4P) on the performance index for energy conservation from photons absorbed by PSII to the reduction of intersystem electron acceptors PI_ABS_.

**Fig 9:**
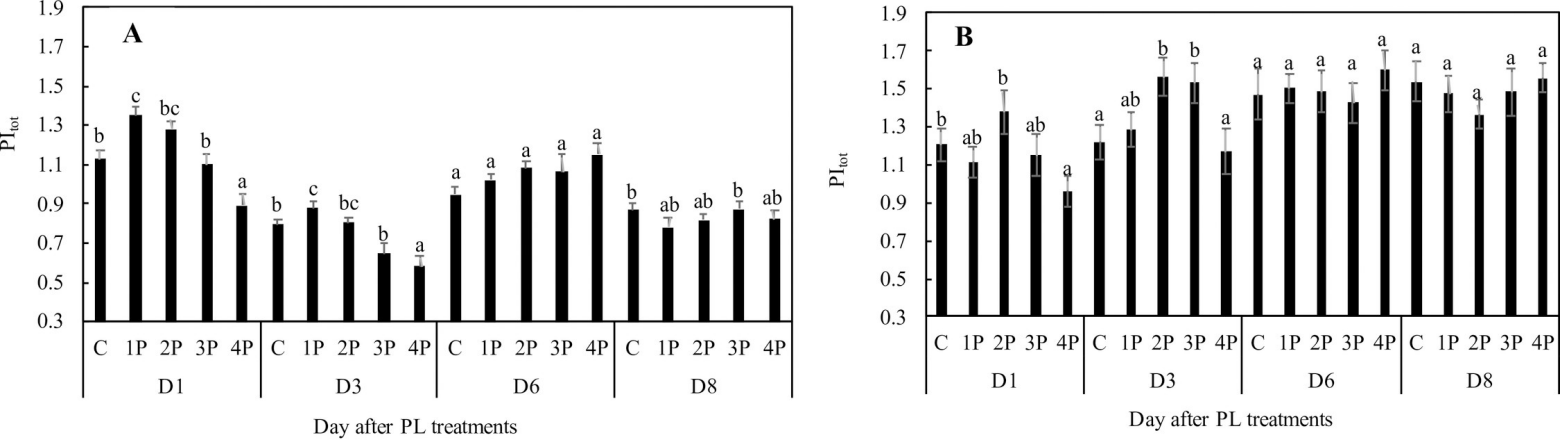
Effects of PL treatments (1P, 2P, 3P, 4P) on the performance index for energy conservation from photons absorbed by PSII antenna until the reduction of PSI acceptors (PI_tot_) measured during trial one **(A)** and trial two **(B)**.

Generally, PI values in all treatments in trial one were within the accepted range for normally performing leaves throughout the trial (Figs 8 and 9A). However, a 14.5% (2.6%) decrease in PI_abs_ (PI_tot_) compared to those of the control was observed in the 3P treatment at D1. This decrease was even more marked in the 4P treatment on the same date: 25.4% for PI_abs_ and 21% for PI_tot_ compared to those of the control (Figs 8 and 9A). In trial two, we similarly observed a 20% decrease in PI_tot_ at D1 as a consequence of the 4P treatment (Fig 9B). Decreases in the PIs can be attributed to decreases in RC/ABS, F_v_/F_0_, (1-V_j_)/V_j_) or RE_0_/ABS (the latter for PI_tot_). At D1, in trial one, we observed no changes from the control in RC/ABS or RE_0_/ABS. F_v_/F_0_ and (1-V_j_)/V_j_) were 15% and 8.2% lower, respectively, than those of the control in the 4P treatment (S1A and S1B Fig). (1-V_j_)/V_j_) was 5.3% lower than that of the control in the 3P treatment. At D3, there were similarly no differences from the control in RC/ABS (S1C Fig), but RE_0_/ABS was 9.8% lower than that of the control in the 4P treatment (S1D Fig) and F_v_/F_0_ was 20% and 29.3% lower in the 3P and 4P treatments, respectively, compared to that of the control (S1A Fig). At D6, there was no longer a noticeable impact of treatments on PIs or their components, suggesting full recovery of the photosynthetic machinery. In trial two there was also a full recovery of PI_tot_ at D8 (Fig 9B). Consistent with the transient positive effect of 1P and 2P treatments on net photosynthetic assimilation (see below), there was even a 19.8% and a 13.4% increase in PI_tot_ at D1 for the 1P and 2P treatments, respectively, compared to that of the control (trial one). This positive effect was still visible at D3 for the 1P treatment. At D1, the effect was attributable to a small increase in F_v_/F_0_ for the 1P treatment, and to a 29% and an 11.3% increase in RE_0_/ABS for the 1P and the 2P treatments, respectively, compared to those of the control. In trial two, we also observed a transient increase in PI_tot_ (27%) for some treatments (2P and 3P) at D3, compared to that of the control (Fig 9B).

**Fig 10:**
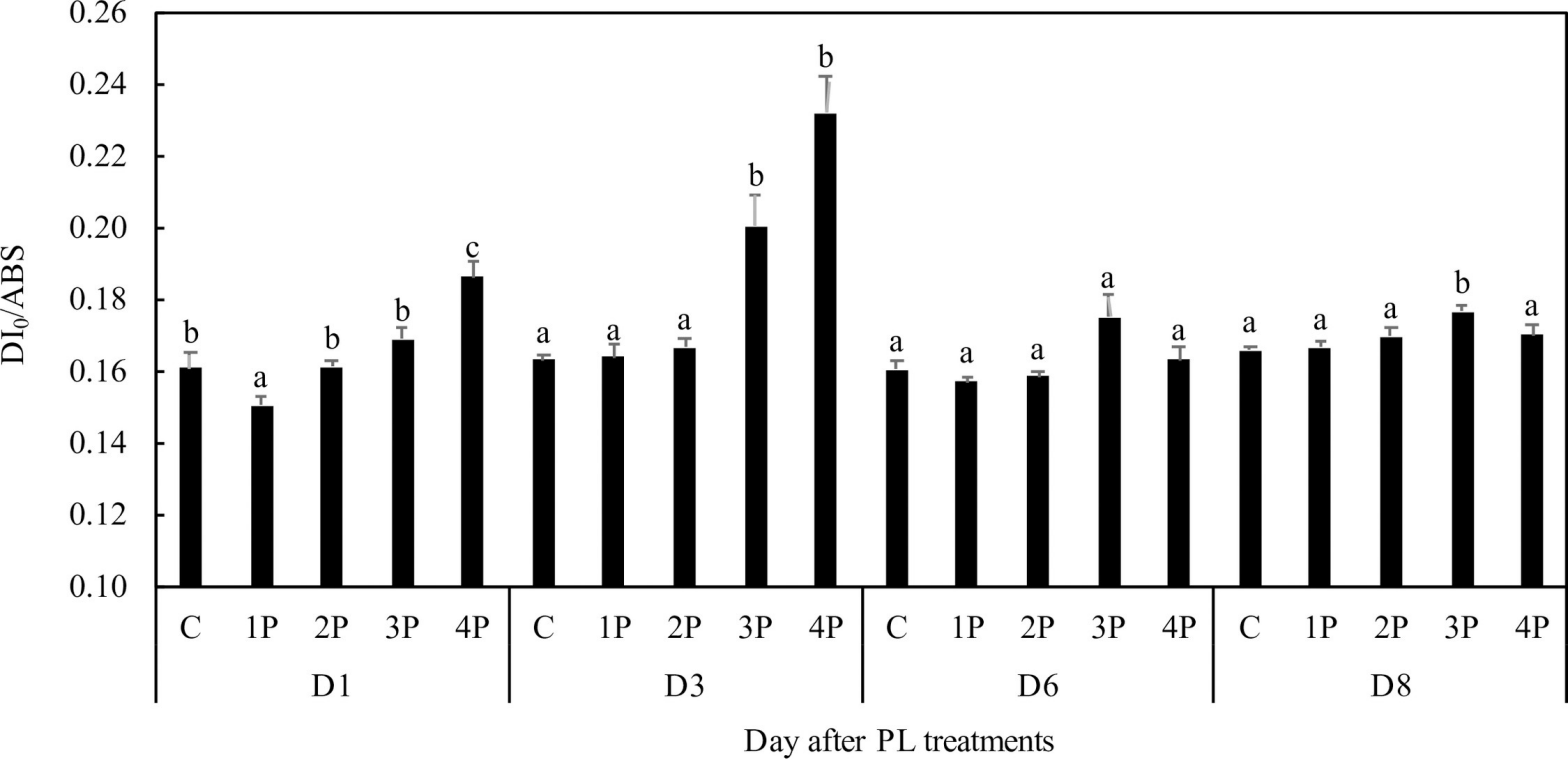
Effects of PL treatments (1P, 2P, 3P, 4P) on the dissipated energy on absorbed photon flux DI_0_/ABS.

In trial one, DI_0_/ABS was 16% higher than that of the control in the 4P treatment at D1 and 42.3% higher at D3 (Fig 10). A 22.3% increase compared to that of the control was observed in the 3P treatment at D3. This increase appeared later but was still apparent at D8 in this treatment, which was not the case in the 4P treatment.

### Effect of PL on leaf net photosynthesis and on leaf maximal photosynthesis

**Fig 11:**
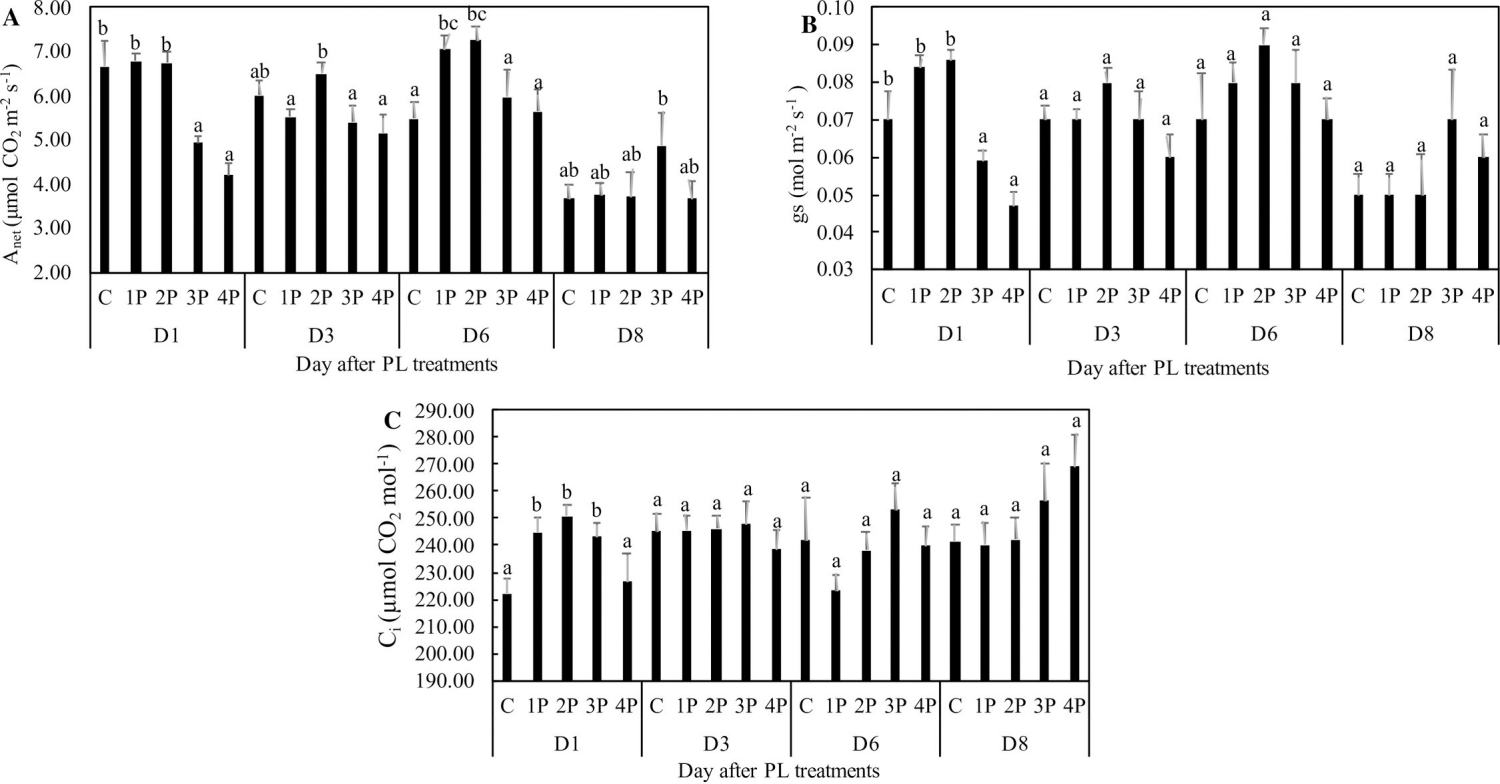
Effects of PL treatments (1P, 2P, 3P, 4P) on leaf net photosynthesis. The measurements were made 1, 3, 6 or 8 days after PL treatments (Trial one). A_net_ represents the net carbon dioxide assimilation **(A)**, g_s_ the stomatal conductance **(B)** and C_i_ the intercellular CO_2_ **(C)**.

The 3P and 4P treatments resulted in 26.1% and a 36.9% decreases, respectively, in A_net_ at D1 compared to that of the control. This negative effect was transient and was no longer visible at D3 (Fig 11A). In contrast to the 3P and 4P treatments, the 1P and 2P treatments resulted in a transient increase in A_net_ when compared to that of the control. At D6, this increase was 28.6% and 32.5%, respectively, in the 1P and 2P treatments. Two days later, at the end of the trial, there were no longer any differences in A_net_ between treated plants and the control. g_s_ was positively correlated with A_net_ throughout the trial (Fig 11B), while C_i_ data were relatively homogeneous (Fig 11C), suggesting homeostasis.

**Fig 12:**
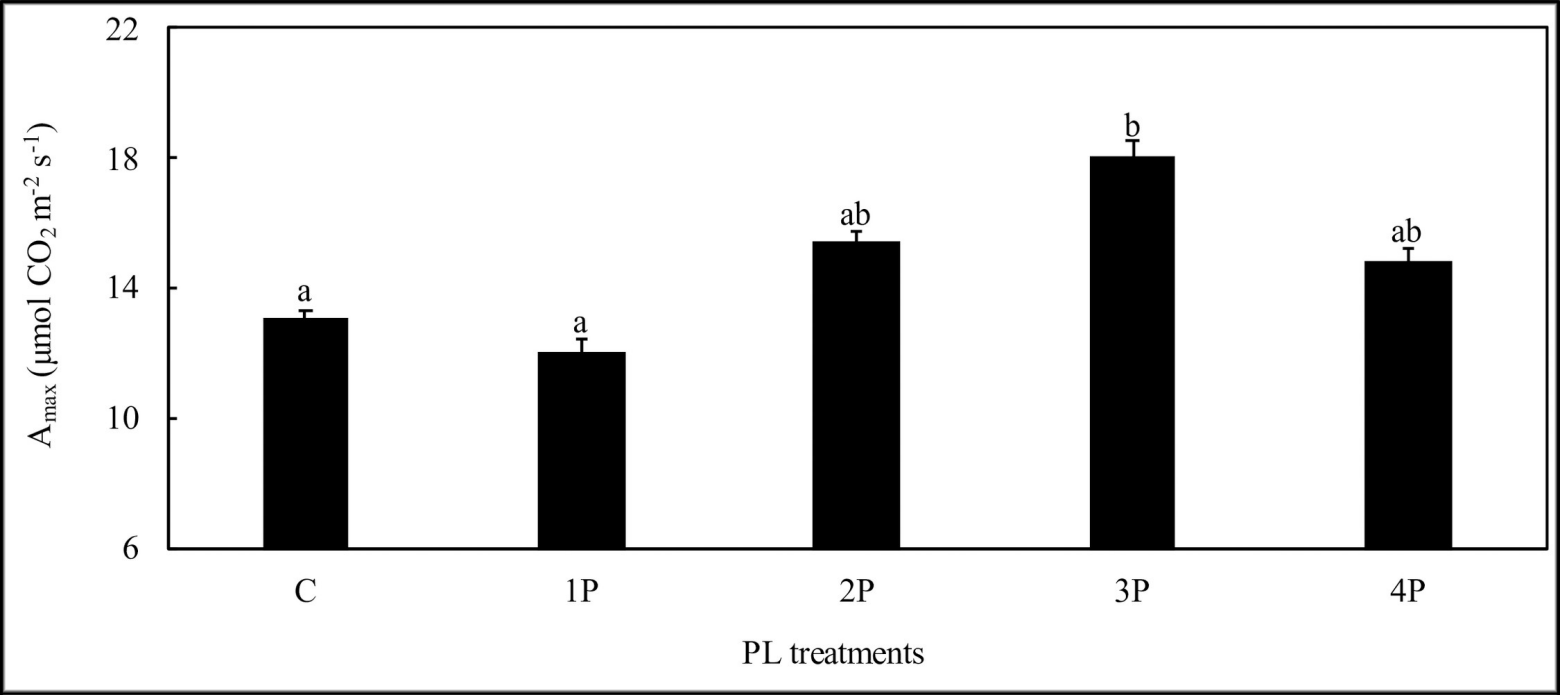
Effects of PL treatments (1P, 2P, 3P and 4P) on leaf maximum net assimilation photosynthesis (A_max_) at D10 (Trial one).

No negative effect on maximal net photosynthetic assimilation (A_max_) was observed in any of the PL treatments. The 3P treatment even resulted in a 38.4% increase in A_max_ compared to that of the control ten days after the light treatment, which is an indication of the positive long-term adaptation of photosynthetic capacity (Fig 12).

## Discussion

### Leaf epidermal flavonols and hydroxycinnamic acids

The hydroxycinnamic acid index was within the expected range [31], but the flavonoid index values were much lower in our trials than in similar trials on lettuce. In one recent trial, [22] found flavonol indexes well above 1 in several cultivars of lettuce grown under direct sunlight. We may therefore attribute the low values we observed in our trials to the early stage of development of the plants and to the lack of UV light, which is a major stimulating factor for the biosynthesis of flavonoids. In greenhouse conditions (trial one) glass is known to block UV light and in growth chambers (trial two), the white light LEDs do not supply UV radiation. In trial one, temperatures were also higher than those in trial two. In general, too high a temperature can inhibit biosynthesis and cause the degradation of flavonoids [32], which may explain why the flavonol index values were lower in trial one than in trial two. Our observations of increases in leaf epidermal flavonols and hydroxycinnamic acids with PL are consistent with similar observations made on the effects of visible light and UV light [22, 30, 33]. Our observations not only confirm previous observations about the potential of PL to increase the phytochemical content of harvested fruits [5, 7– 9, 34], but they also suggest that PL could be used on entire growing plants to stimulate secondary metabolism in greenhouses or in field conditions. However, assessing whether PL is harmless to plants is a prerequisite.

### Damage indicators

We followed here the ChlF-based method for potential damage assessment of [23]. F_v_/F_m_ values average approximately 0.83–0.84 in most nonstressed C3 plants [35, 36]. While slightly lower values of F_v_/F_m_ can be interpreted in terms of photoprotection, substantially lower values of F_v_/F_m_ are indicators of photodamage [37]. Similarly, higher values of F_0_ can suggest damage [38]. In trial one, F_0_ and, as a consequence, F_v_/F_m_ values, suggest that the damaging effect of PL is all the more substantial and swift when there are more pulses. It was not until D3 that a relatively substantial decrease in F_v_/F_m_ could be observed, and then only in the 4P and, to a lesser extent, in the 3P treatments. Eight days after treatment, there were no longer any differences from the control in any of the PL treatments with the exception of the 3P treatment. In the 3P treatment on D8, F_v_/F_m_ was only slightly lower than that of the control. In trial two, the 2P, 3P and 4P treatments also showed increases in F_0_, depending on the date, but the differences were much less pronounced than those in trial one. Moreover, associated decreases in F_v_/F_m_, whenever observable, such as in 2P at D8, were less pronounced than those in trial one. We may attribute these differences in results between trial one and trial two to the fact that temperature was controlled in the latter, suggesting that the effect of PL treatments on F_0_ and F_v_/F_m_ is temperature-dependent. As a whole, F_v_/F_m_ data suggest that single treatments of up to four pulses can have damaging effects on the photosynthetic machinery, but the effects are only moderate and transient ones. This conclusion is supported by the V_k_/V_j_ and S_m_ data.

Limitation/inactivation, possibly through damage to the oxygen-evolving complex (OEC), may be observed and assessed through the increase in V_k_/V_j_ [39, 40]. A K-step occurs whenever the electron flow to the acceptor side exceeds the electron flow from the donor side. This leads to RC oxidation with a photosystem shift towards the P680+ form, which is known to have a low ChlF yield [39]. Thus, OEC dissociation triggers the K-step, by inhibiting efficient electron donation to the RC [39, 41]. Increased V_k_/V_j_ values suggest that some limited but noticeable damage to the OEC may have occurred after a certain delay (from D3). The dynamics of V_k_/V_j_ were not very different from the dynamics of F_0_, with a full recovery for all treatments observable at D8, with the exception of the 3P treatment in trial one. In trial two, there was no longer an observable effect at D8 for any of the four PL treatments, suggesting that the negative effect of PL treatments was even less pronounced under controlled temperature conditions.

S_m_ is assumed to be proportional to the pool size of electron carriers, and decreases in S_m_ are suspected to be indicators of stress-associated damage [21, 42, 43]. We observed a transient increase in S_m_ at D1 in all four treatments of trial one compared to that of the control, but there was a decrease in the 2P treatment at D3 and in the 1P and 4P treatments at D8. In trial two as in trial one, there was an increase in S_m_ for the 3P treatment at D8. It is difficult to draw a clear conclusion from such observations, but the results suggest that a moderate effect of PL possibly even a positive effect, can still be present 8 days after the treatments, at least for some treatments.

As a whole, our observations suggest that PL had some damaging, or at least inhibiting effects on the photosynthetic machinery. Such effects generally seem to appear earlier and to be more pronounced with the increasing number of the pulses. The effects also seem to be more pronounced in the absence of temperature control (trial one). However, after 8 days, there was a near- to- complete recovery in all PL treatments, and possibly a positive effect of some treatments (3P) on the plastoquinone pool.

### Performance indicators and parameters related to the rerouting of energy and electron fluxes

As a multiparametric variable integrating RC/ABS, F_v_/F_0_ and (1-V_j_)/V_j_, PI is a much more sensitive and discriminating stress indicator than F_v_/F_m_ [44]. Its decrease for certain dates and treatments was indeed more pronounced than the decrease in F_v_/F_m_; see, for instance, the 3P and 4P data on D3 (trial one). In PI_abs_, RC/ABS represents the contribution of the density of active reaction (in the sense of quinone acceptor (Q_A_) reducing) centers (on a chlorophyll basis), F_v_/F_0_ represents the contribution to PI of light reactions for primary photochemistry, i.e., the performance due to the probability of trapping excitation energy, and (1-V_j_)/V_j_ represents the contribution of dark reactions to PI, i.e., the performance due to the conversion of excitation energy into photosynthetic electron transport. The lower PI_tot_ values found for the 4P treatments at D1 were attributable to a decrease in F_v_/F_0_ and (1-V_j_)/V_j_. A decrease in RC/ABS reflects the downregulation of PSII reaction centers, a well-known mechanism of light adaptation in leaves [45], but we did not observe a decrease in RC/ABS as a consequence of PL in our trial, which indicates that PL does not impact the photosynthetic machinery in the same way as a sudden exposure to high light. The decrease in F_v_/F_0_ suggests that the probability of trapping excitation energy was reduced, which is consistent with the observations made in coffee leaves subjected to increased photon flux density [25]. The decrease in F_v_/F_0_ was associated with a substantial increase in DI_0_/ABS at D3 in the 4P treatment. Changes in DI_0_/ABS reflect changes in dissipation, mainly as heat, of excess absorbed energy. An increase in energy dissipation is expected to be associated with reduced trapping of excitation energy [25]. The decrease in (1-V_j_)/V_j_ values we observed may be interpreted as the consequence of a reduced ability to process NADPH, which would impair electron transport capacity on the PSII acceptor side. Again, this decrease is consistent with the observations made by [25]. The lower values of RE_0_/ABS in the 4P treatment at D3 suggest that the electron transport capacity was further impaired, beyond the PSII acceptor site, to the PSI acceptors. In contrast, there was a transient increase in RE_0_/ABS in the 1P and 2P treatments at D1.

The values of PIs (both PI_abs_ and PI_tot_) and their components are consistent with the values of the damage parameters; they basically confirm that the 4P treatment and, to a lesser extent, the 3P treatment have transient negative effects that trigger adaptative mechanisms. They also show that transient positive effects can be observed with some treatments, a fact confirmed by trial two.

### Leaf net photosynthesis and photosynthetic capacity

Clearly, PL not only impacted electron and energy fluxes in and around photosystems, but also influenced A_net_. While the 3P and 4P treatments reduced A_net_ one day after treatments, the 1P and 2P treatments exerted a positive effect, observable at D6 (trial one). The transient negative effect of PL for the 3P and 4P treatments does not seem attributable to a negative effect on g_s_ since C_i_ was not reduced. Similarly, the transient positive effect of the 1P and 2P treatments at D6 is not attributable to a positive effect on g_s_. The fact that g_s_ and A_net_ values appear to be correlated must be interpreted as the consequence of coregulation of these parameters [46]. In our trial, g_s_ changed as a consequence of changes in A_net_, not vice versa. The gas exchange data are not fully consistent with the ChlF data. The decrease in A_net_ in the 3P and 4P treatments at D1 is consistent with the decrease in PI_abs_ on the same day, but the increase in A_net_ in the 1P and 2P treatments at D6 is not reflected in the PIs data.

The effect of light exposure on leaf photosynthetic capacity is well documented [47]. However, light flashes supplied at a given time are very different from increased exposure to high PPFD for extensive periods of time. It was therefore very surprising to find that photosynthetic capacity (A_max_) was increased in the 3P treatment, 10 days after plants were irradiated. It is certainly necessary to confirm this effect in the future and then analyze it, to determine whether it is due to an increase in the maximum carboxylation rate, the light-saturated rate of electron transport or triose-phosphate utilization [48–50]. Additionally, it would be necessary to analyze the relationship between photosynthetic capacity and leaf nitrogen content [47].

## Conclusion

Our results, obtained on lettuce leaves, show that hormetic doses of PL, i.e., doses that, in this case, are capable of driving secondary metabolism without causing negative side effects to photosynthesis, can be defined for greenhouse conditions. Our results clearly represent a first incentive to consider PL in addition to pure UV-C light for greenhouse or field use in the future. PL could be tested in greenhouse and field conditions with the objective of increasing the phytochemical content of fruits and vegetables and also possibly of crops for the cosmetic, pharmaceutical and food industries. Of course, security issues will have to be treated satisfactorily. In addition, it is important to assess and understand better the systemic effects of PL since that will determine the size of lamps that would be used in crop canopies in the field. On the scientific side, studies must be conducted in the future with the objective of better understanding the effects of PL. The biological effects of PL have been attributed principally to the UV radiation it supplies, notably the UV-C radiation. However, the other components of PL may also play a role. Complementary, synergetic or antagonistic effects could exist between the different wavelengths that make up the PL spectrum. In addition, as the effect of certain PL treatments on A_max_ suggests, more studies are needed to obtain a better view of the full range of the biological effects of PL, and a better understanding of their underlying physiological mechanisms.

## Supporting information

S1 Fig Effects of PL treatments (1P, 2P, 3P, 4P) on parameters of the the perfermance index. The measurments were made 1, 3, 6 or 8 days after PL treatments (Trial one). Fv/F0 the contribution to the PI of the light reactions for primary photochemistry (A), (1-Vj)/Vj the performance due to the conversion of excitation energy to photosynthetic electron transport (B), RC/ABS the density of active PSII reaction centers expressed on the base of the quantity of light absorbed by the antenna (C) and RE0/ABS the electron transport flux from QB to PSI acceptors, RE, expressed as quantum yield (/ABS) (D).
(TIF)

S1 File Data of flavonol index, hydroxycinnamic acids and fluorescence parameters. (ZIP)
