# Supplementary figures and images for: Xenon lamps used for fruit surface sterilization can increase the content of total flavonols in leaves of Lactuca sativa L. without any negative effect on net photosynthesis
Source: PLoS One. 2019 Oct 21;14(10):e0223787. doi: 10.1371/journal.pone.0223787 (PMC6802843; doi:10.1371/journal.pone.0223787)

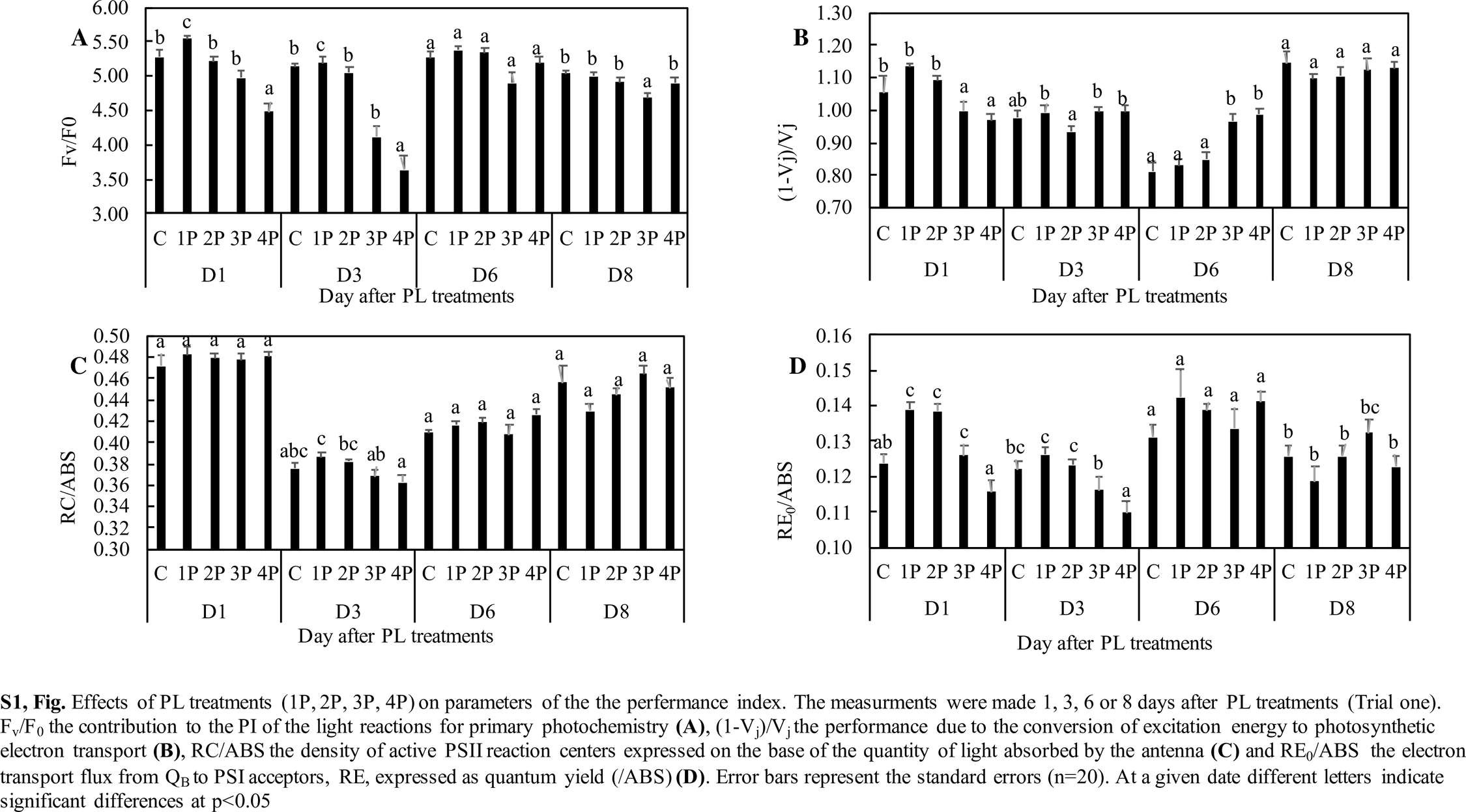

Supplement: S1 Fig — The measurments were made 1, 3, 6 or 8 days after PL treatments (Trial one). Fv/F0 the contribution to the PI of the light reactions for primary photochemistry (A), (1-Vj)/Vj the performance due to the conversion of excitation energy to photosynthetic electron transport (B), RC/ABS the density of active PSII reaction centers expressed on the base of the quantity of light absorbed by the antenna (C) and RE0/ABS the electron transport flux from QB to PSI acceptors, RE, expressed as quantum yield (/ABS) (D). (TIF) [file pone.0223787.s001.tif]
